# Covalent Functionalization of Graphene Oxide with Fructose, Starch, and Micro-Cellulose by Sonochemistry

**DOI:** 10.3390/polym13040490

**Published:** 2021-02-04

**Authors:** María Montserrat Cruz-Benítez, Pablo Gónzalez-Morones, Ernesto Hernández-Hernández, José Roberto Villagómez-Ibarra, Javier Castro-Rosas, Esmeralda Rangel-Vargas, Heidi Andrea Fonseca-Florido, Carlos Alberto Gómez-Aldapa

**Affiliations:** 1 Instituto de Ciencias Básicas e Ingeniería, Universidad Autónoma del Estado de Hidalgo, Ciudad del Conocimiento, Carretera Pachuca—Tulancingo km 4.5, C.P. 42184 Mineral de la Reforma, Mexico; montserrat9004@gmail.com (M.M.C.-B.); jrvi@uaeh.edu.mx (J.R.V.-I.); jcastro@uaeh.edu.mx (J.C.-R.); esme_ran70@hotmail.com (E.R.-V.); 2 Centro de Investigación en Química Aplicada (CIQA), Boulevard Enrique Reyna Hermosillo, No. 140, C.P. 25294 Saltillo, Mexico; pablo.gonzalez@ciqa.edu.mx (P.G.-M.); ernesto.hernandez@ciqa.edu.mx (E.H.-H.); 3 CONACYT, Centro de Investigación en Química Aplicada (CIQA), Blvd. Ing. Enrique Reyna H. No. 140, C.P. 25294 Saltillo, Mexico

**Keywords:** nanohybrids, graphene oxide, organic molecules, sonication, ultrasound

## Abstract

In this work, we report the synthesis of graphene oxide (GO) nanohybrids with starch, fructose, and micro-cellulose molecules by sonication in an aqueous medium at 90 °C and a short reaction time (30 min). The final product was washed with solvents to extract the nanohybrids and separate them from the organic molecules not grafted onto the GO surface. Nanohybrids were chemically characterized by Fourier-transform infrared spectroscopy (FTIR), X-ray photoelectron spectroscopy (XPS), and Raman spectroscopy and analyzed by thermogravimetric analysis (TGA), scanning electron microscopy (SEM), and X-ray diffraction (XRD). These results indicate that the ultrasound energy promoted a chemical reaction between GO and the organic molecules in a short time (30 min). The chemical characterization of these nanohybrids confirms their covalent bond, obtaining a grafting percentage above 40% the weight in these nanohybrids. This hybridization creates nanometric and millimetric nanohybrid particles. In addition, the grafted organic molecules can be crystallized on GO films. Interference in the ultrasound waves of starch hybrids is due to the increase in viscosity, leading to a partial hybridization of GO with starch.

## 1. Introduction

The use and application of graphene has a significant impact in several science and technology fields since they take advantage of its excellent mechanical, electrical, and optical properties [1,2,3]. In many of these applications, graphite is initially oxidized to produce graphite (GrO) and graphene oxide (GO) [4]. These graphemic materials are chemically grafted or covalently functionalized with other organic and inorganic molecules (nanohybrids) [5] to be used as raw material in the manufacturing of solar cells [6], battery electrodes [7], biosensors [8], and prostheses [9], among others [10,11], where graphite and GO considerably improve the performance of the products. Nanohybrids grafted with organic molecules, as starch and fructose, are used due to their biodegradation [12,13] and biocompatibility as material for antibiotic recovery in milk [14], manufacturing of antimicrobial films for foods [15,16], formulation of bio-nano-compounds [17,18,19,20,21,22], supercapacitors [23,24,25,26], fuel cells [27], water purification membranes [28,29,30], biosensors [31,32], adhesives [33], and bone implants [34]. To synthetize these GO/starch [35,36,37,38,39,40,41,42,43,44,45,46] and GO/fructose [27] nanohybrids, chemical processes include exfoliated graphite by sonication in an aqueous medium or polar solvent for several hours. Starch or fructose are added and mixed at 30–180 °C for 9–72 h (reaction time) [25,35,43]. Other methods use solvents as acetonitrile [27], dimethylformamide [40], and anhydrous dimethylsulfoxide [44] along with other additives to control pH (sodium hydroxide, ammonia) [38,46], reduce reaction time by 4–6 h (hydrazine, epichlorohydrin) [37,38], and activate hydroxyl groups (OH) of GO (1-ethyl-3-(3 dimethylaminopropyl) carbodiimide chlorohydrate, 4-dimethylaminopyridine) [44].

Considering the above, due to the importance and possible applications of nanohybrids with organic molecules, it is necessary to develop new and environmentally-friendly processes and technologies. This could be achieved by reducing the use of solvents, high temperatures, and reaction times, without affecting nanohybrid properties (degree of exfoliation and hybridization) or GO chemical degradation. That is why the present research work developed a process for the covalent functionalization (hybridization) of GO with starch (S), fructose (F), and microcrystalline cellulose (M) molecules. Sonochemistry is applied while the reaction time and the use of polar solvents are significantly reduced, without compromising or negatively affecting the final characteristics of the nanohybrids. The ultrasound energy is key to promote the chemical reaction between the functional groups of GO and S, F, and M molecules, which considerably reduces reaction times (30 min). Then, we obtained nanohybrids with grafting from 41.08% to 63.99% weight of S, F, and M. This high concentration of organic molecules prevents GO sheets from stacking, as it happens with graphite. Additionally, certain amount of S, F, and M molecules are crystallized on the GO surface. Given these results, reaction times in this work are reduced and the creation of films using these nanohybrids is promoted.

## 2. Materials and Methods

### 2.1. Materials

D-Fructose (F) and microcrystalline cellulose (M) (particles measuring ~50 µm) were obtained from Sigma-Aldrich Co (St. Louis, MO, USA). Cassava starch (S) was purchased from Royal Ingredients Group B.V. (Alkmaar, Holland). All reagents were of an analytical grade unless specified. Following Hummers method with the variation proposed by Hernández-Hernández et al. [47], graphite was prepared in the laboratory using graphite powder from Graftech (particle size < 75 μm) as raw material.

### 2.2. Preparation of Organic Molecules

Three synthetic routes were established to synthesize the GO grafts with starch (S), fructose (F), and micro-cellulose (M). An aqueous graphite solution (0.05 g/25 mL water) was kept in constant agitation for 12 h. The solution was then placed in an ultrasound bath for 10 min to increase the interlaminar distance of the graphite sheets. Separately, 0.25 g F was dissolved in 25 mL of water and kept under constant agitation. A pre-gelatinized solution was obtained from a starch slurry. In addition, 5 g of S was dispersed in 100 mL of water and placed in a water bath at 90 °C for 10 min. A NaOH solution (26.9 mL, 11%) was used to dissolve 0.5 g of M and frozen for 12 h. Subsequently, it was thawed at room temperature and 20.6 mL of distilled water was added.

### 2.3. Graft Synthesis of GO Whit Starch, Fructose, and Micro-Cellulose

**Scheme 1:**
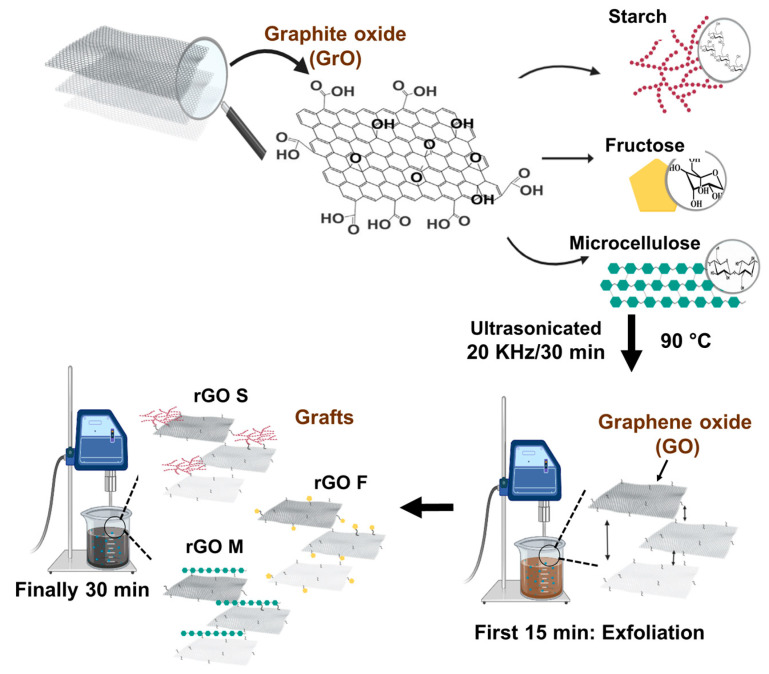
Ultrasound-assisted graft synthesis process.

Scheme 1 shows graft formation. F, S, or M were added to the graphite solution while stirring. Each mixture was ultrasonicated in an ultrasonic processor (CPX750, Cole-Parmer, Bunker Ct, Vernon Hills, IL, USA) at 750 W, 20 kHz, and 80% amplitude for 30 min. During sonication, the sample was kept in a water bath at 90 °C. The mixture formed a brown suspension (original color of the graphite) and became completely black after 15 min, as shown in the supplementary information (Figure S1). The color changes due to gas release, which suggests the chemical reaction of graphite with the release of CO_2_ and water. The homogeneity and coloration of the suspension indicate the reduction of GO. Each sample was then washed several times with hot distilled water and centrifuged at 4500 rpm and 25 °C for 30 min until the supernatant showed a transparent color. The sample was dried out in an oven with air flow at 80 °C for 12 h. Then, samples were pulverized and stored at room temperature until further analysis. The purified grafts are referred to as rGO F (fructose), rGO S (starch), and rGO M (micro-cellulose).

### 2.4. Characterization

The interactions between functional groups of rGO F, rGO S, and rGO M were assessed using different techniques. For the characterization by Fourier-transform infrared spectroscopy (FTIR), a spectrometer (IS5, Thermo Scientific, Waltham, MA, USA) was used. The total reflectance was attenuated (PIKE Technologies, Fitchburg, WI, USA) from 4000 cm^−1^ to 400 cm^−1^ with 120 scans. A Raman spectroscopy analysis was performed (Micro-Raman XploRA, Horiba, Kyoto, Japan) at a frequency range of 1000–400 cm^−1^ using a 532-nm laser source. An X-Ray photoelectron spectroscopy (XPS) analysis was performed in an X-ray photoelectron spectrometer (PHI 5000 VersaProbe II XPS) with a monochromatic Al anode (1486.6 eV) as the X-ray source. The survey spectra were obtained with a pass energy of 117.5 eV while the analysis region was 1400–0 eV. The XPS signal was obtained with a pass energy of 11.5 eV. A dual beam charge neutralization system (PHI-patented) was used to compensate for charging during XPS data acquisition. All measurements were made in an ultra-high vacuum (UHV) chamber at an approximate pressure of 3 × 10^−8^ mbar. X-Ray diffraction (DRX) was performed in an X-ray diffractometer (D8 Advance ECO, Bruker, Billerica, MA, USA) with Kα radiation from Cu (1.5418 Å) at 2θ = 5 to 40° connected to a power supply of 40 kV and 25 mA. A thermogravimetric analysis (TGA) was performed with a thermogravimetric analyzer (TA-Q500, TA Instruments, New Castle, DE, USA) from 25 to 600 °C under continuous nitrogen flow (50 mL/min) and a heating rate of 10 °C/min. The graft morphology was observed under a scanning electron microscope (SEM) (IT-300, JEOL, Akishima, Japan) at 15 A and 20 kV.

## 3. Results

The nanohybrids, purified and washed by centrifugation, were characterized and analyzed. We used FTIR, XPS, Raman, and TGA to assess rGO S, rGO F, and rGO M.

**Figure 1:**
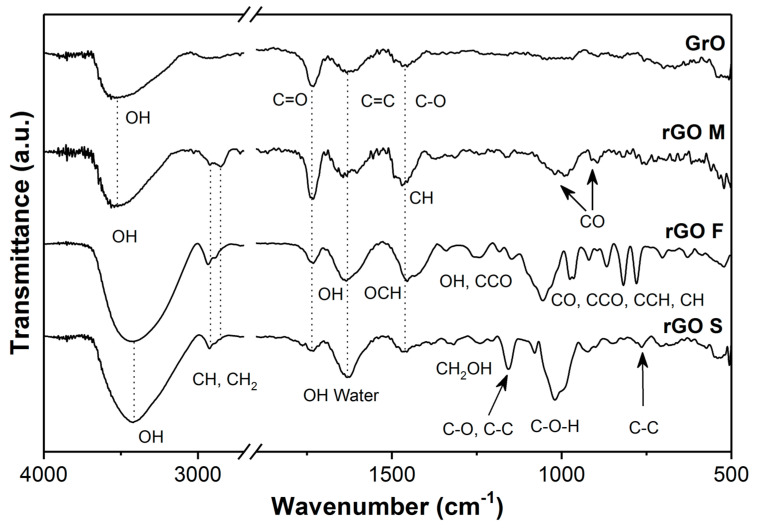
Fourier-transform infrared spectroscopy (FTIR) of graphite (GrO) and GO nanohybrids with starch (GO S), fructose (GO F), and micro-cellulose (GO M).

**Figure 2:**
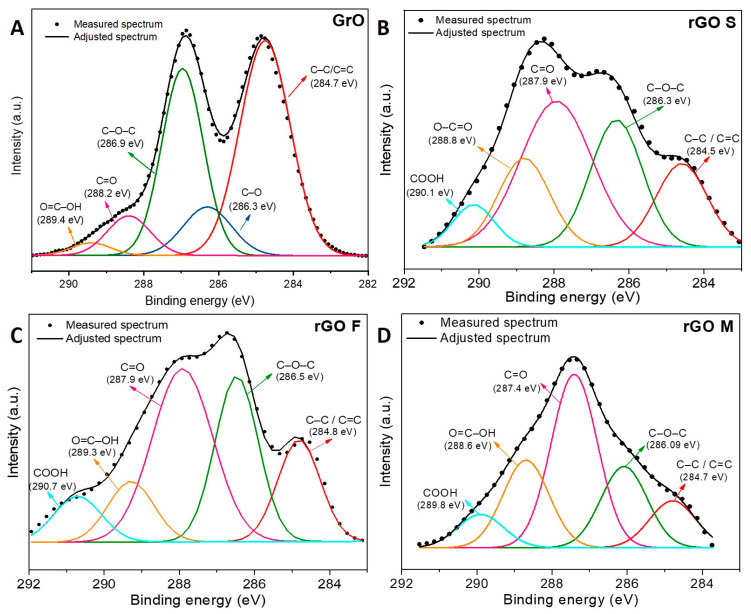
High-resolution X-ray photoelectron spectra (XPS) spectra of (**A**) graphite (GrO), (**B**) rGO S, (**C**) rGO F, and (**D**) rGO M.

Figure 1 shows FTIR spectra of graphite and nanohybrids, where graphite shows characteristic signals of OH functional groups at 3520 cm^−1^, C=O at 1735 cm^−1^, aromatic C=C at 1629 cm^−1^, and C–O at 1460^−1^ [48,49]. However, nanohybrids showed two significant changes after sonication: the presence of new signals and increased intensity in signals already identified in graphite. The new signals in rGO M are attributed to functional groups characteristic in micro-cellulose [50]: CH/CH_2_ at 2918 and 2854 cm^−1^ and C–O at 891–1017 cm^−1^, and increased intensities at 1452 and 1735 cm^−1^, corresponding to C–H and C=O groups of micro-cellulose. In rGO F, the new peaks are common in fructose [51]: 2926, 2878 cm^−1^ for CH/CH_2_, 1247 cm^−1^ for OH/CCO, and 1054–625 cm^−1^ for CO, CCO, CCH, CH_2_, and CH. In addition to an increase at 3416 and 1630 cm^−1^ attributed to the OH group and 1460 cm^−1^ for the OCH group in fructose. In GO S, new signals characteristic in starch are shown [52]: CH_2_OH at 1240 cm^−1^, C–O/C–C at 1153 cm^−1^, CH at 1016 cm^−1^, C–O–C at 920 cm^−1^, and C–C at 761 cm^−1^, and an increase at 3416 and 1630 cm^−1^ due to starch. These FTIR studies indicate that, after sonication, GO showed changes in its chemical composition while additional signals were identified in all the hybrids, corresponding to the organic molecules with which they were sonicated. Therefore, micro-cellulose, fructose, and starch molecules are deposited or grafted on GO. To prove this chemical alteration, XPS studies were carried out, as shown in Figure 2 and Table 1.

Figure 2A shows the high-resolution XPS spectra for 1 s in each of the samples and their respective deconvoluted signals. Table 2 presents the characteristic energies of the functional groups present as well as the percentage corresponding to each sample. In graphite, the highest percentage of functional groups corresponds to C–C/C=C at 46.8% and C–O–C at 33.5%, corresponding to the intensity of the two peaks in the spectrum in Figure 2A. Still, the nanohybrid spectra show shape and intensity changes in the signals of their functional groups when compared against graphite spectrum. In rGO S, the peak with the highest intensity is that of C=O, followed by C–O-–C, which agrees with the 38.7% and 24.7% values, respectively.

The highest intensity in rGO F is shown by C=O and C–O–C at 38.7% and 24.7%, respectively, and C=O and O=C–OH at 44.5% and 22.4%, respectively, in rGO M. These changes are due to the presence of organic molecules, starch, fructose, and micro-cellulose in the nanohybrids, diluting or reducing the concentration of C–C/C=C in graphite. Contrastingly, the significant increase in C=O (from 7.4 to 44.5%) and O=C–OH (from 2.1 to 22.4%) in the nanohybrids is the result of organic molecules oxidized by sonication and hybridization [53,54]. In consequence, the relation C–C/C=O is reduced from 6.3 to 0.1% in graphite and rGO M.

**Figure 3:**
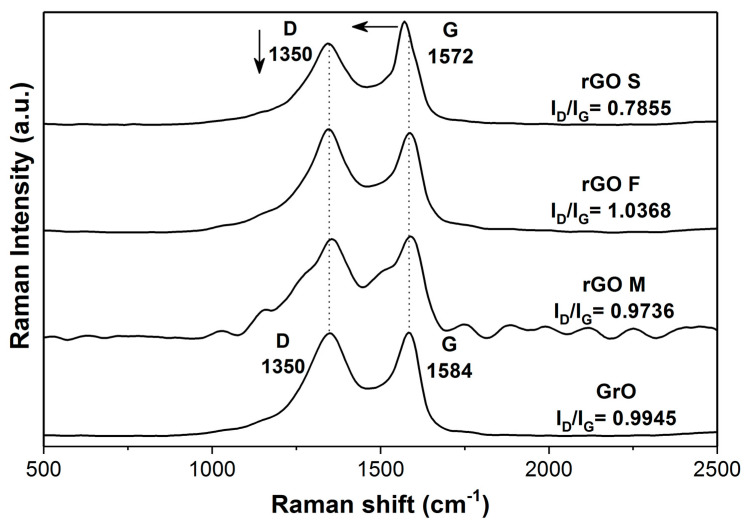
Raman spectra of graphite (GrO) and rGO S, rGO M, and rGO F nanohybrids.

Additionally, the XPS of the nanohybrids indicates the formation of a new peak between 289.1 and 290.7 eV. It represents a clear evidence of the covalent bond between GO and starch, fructose, and micro-cellulose molecules by esterification [55] between the COOH groups of GO and the OH of the organic molecules promoted by ultrasound energy. However, this energy can create defects or vacancies in the hexagonal nanohybrid network. To prove that, Raman spectroscopy was performed as shown in Figure 3.

The Raman studies indicate that graphite shows characteristic bands: D at 1350 cm^−1^ representing sp^3^ hybridization of carbon, related to defects in the hexagonal network of graphite, and G at 1584 cm^−1^ created by the tangential vibration of sp^2^ hybridized carbon atoms and related to graphite purity. The intensity ratio of both bands (I_D_/I_G_) is 0.9945 cm^−1^. Bands D and G of rGO M and rGO F nanohybrids show no displacement and their I_D_/I_G_ ratios are 0.9736 and 1.0368 cm^−1^, respectively. They also show a small absolute difference in relation to graphite (0.0209 and 0.0423, respectively). These results suggest that, during aqueous sonication, a chemical reaction occurs between GO and micro-cellulose and fructose without altering the hexagonal network of GO. In contrast, two changes are observed in rGO S. There is a displacement toward higher values in G, when compared against graphite (from 1584 to 1572 cm^−1^), and a reduction in both D intensity and the I_D_/I_G_ ratio, which is lower than that of graphite by 0.209. These results indicate that GO reaction and reduction with starch molecules take place simultaneously during sonication [39,44].

**Figure 4:**
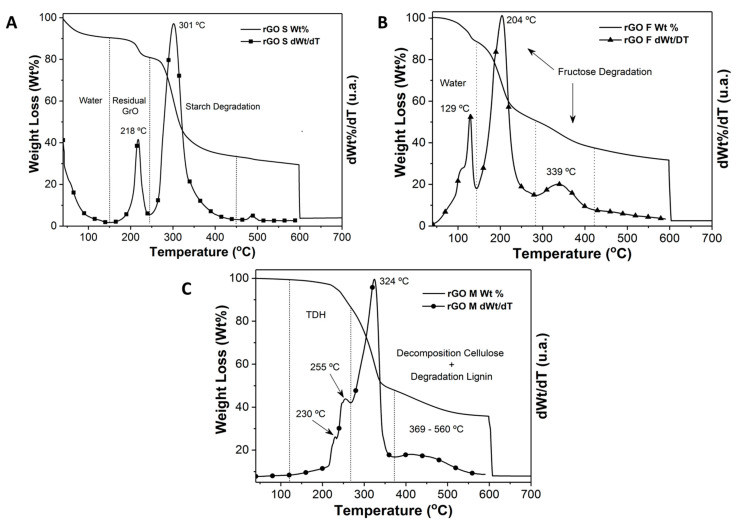
TGA of (**A**) rGO S, (**B**) rGO F, and (**C**) rGO M nanohybrids.

To identify the amount of starch, micro-cellulose, and fructose grafted onto the nanohybrids, TGA was carried out (Figure 4). The TGA corresponding to graphite, starch, fructose, and micro-cellulose are shown in Figure S2 of the Supplementary Information. Thermal degradation is observed in rGO S (Figure 4A), where graphite functional groups (OH, C–O–C) are still found in the peak at 218.9 °C [56] and starch degradation at 301.8 °C [57]. Figure 4B presents the two main peaks of rGO F at 204.5 and 339.1 °C, which are both reported as thermal degradation of the fructose grafted onto carbon nanostructures [58,59,60,61,62]. In both rGO S and rGO F weight loss at temperatures below 150 °C increased considerably (water evaporation) due to the presence of polar groups of organic molecules increasing water absorption in the nanohybrids. No graphite-related weight loss is observed in rGO M (Figure 4C). Still, there are two shoulders at 230 and 255 °C associated with hemicellulose depolymerization. The main peak at 324.4 °C and a smaller one between 369.4 and 560.6 °C correspond to cellulose and lignin decomposition and degradation, respectively [63].

Table 2 shows thermal degradation data of the nanohybrids, graphite weight, and organic molecules grafted onto the nanohybrids. The latter were grafted with 41.08–63.99% weight of organic molecules. See Table S1 of Supplementary Information to identify differences.

We observe that rGO S still shows GO functional groups due to the complexity of the starch reaction system. Since its molecular weight is high, this molecule increases the viscosity of the reaction medium, partially absorbing the ultrasound energy. This hampers the exposure of functional groups in both components and, thus, prevents the reaction of all the GO functional groups with starch and promotes GO reduction.

**Figure 5:**
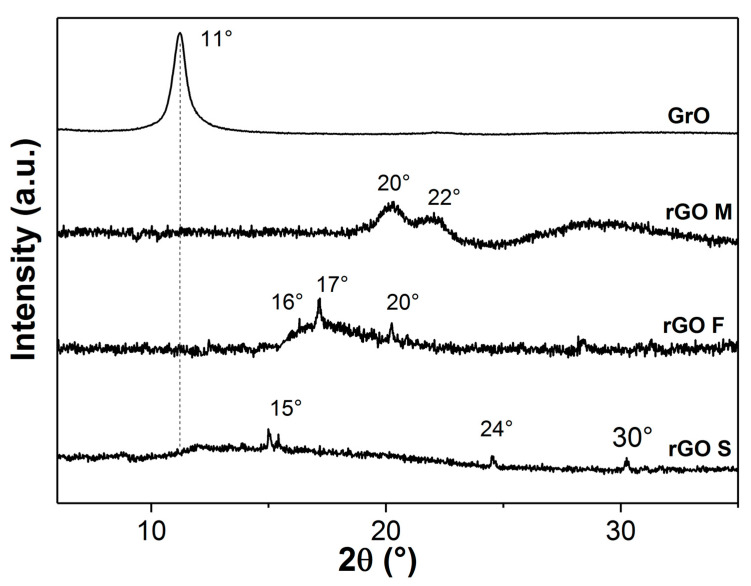
X-ray diffraction patterns of graphite (GrO), rGO S, rGO F, and rGO M nanohybrids.

The morphology of the nanohybrids was analyzed using XRD and SEM. In diffractograms of Figure 5, graphite shows a peak at 2θ = 11.4° representing the distance (0.78 nm) created by the stacking of GO sheets associated with the (001) plane. Still, the nanohybrids do not show this characteristic peak of graphite after sonication. Each nanohybrid shows characteristic peaks of the grafted organic molecules. While rGO S shows three signals at 2θ = 15.1, 24.5, and 30.29°, corresponding to starch [64,65], rGO F exhibits signals at 2θ = 16.3, 17.1, and 20.2°, associated with fructose [66], and rGO M shows two peaks at =20.2 and 22°, belonging to cellulose II [67]. These results indicate that the number of organic molecules chemically grafted onto GO sheets (41.08–63.99% in weight) is so significant that they are crystallized on the surface.

**Figure 6:**
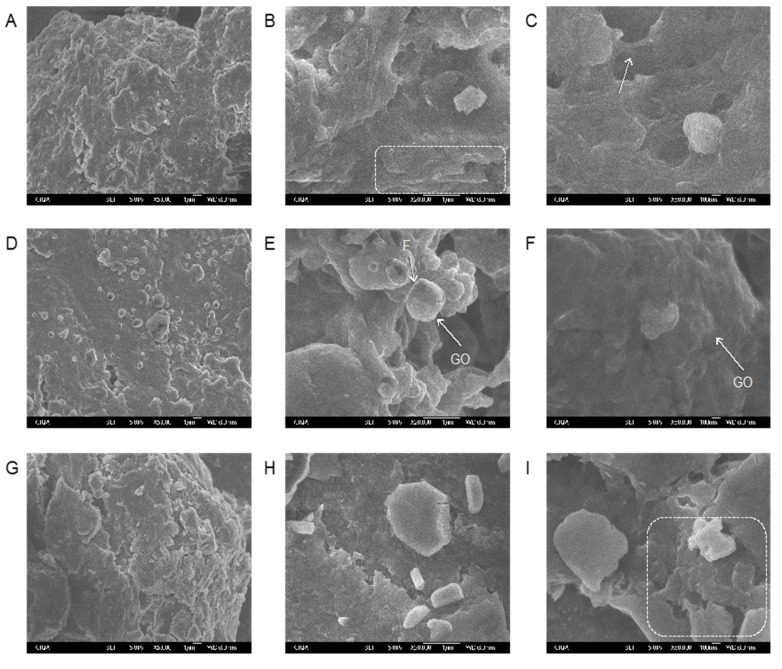
Scanning electron microscopy (SEM) micrographs of rGO S (**A**–**C**), rGO F (**D**–**F**), and rGO M (**G**–**I**) nanohybrids at 5000, 20,000, and 50,000×.

**Figure 7:**
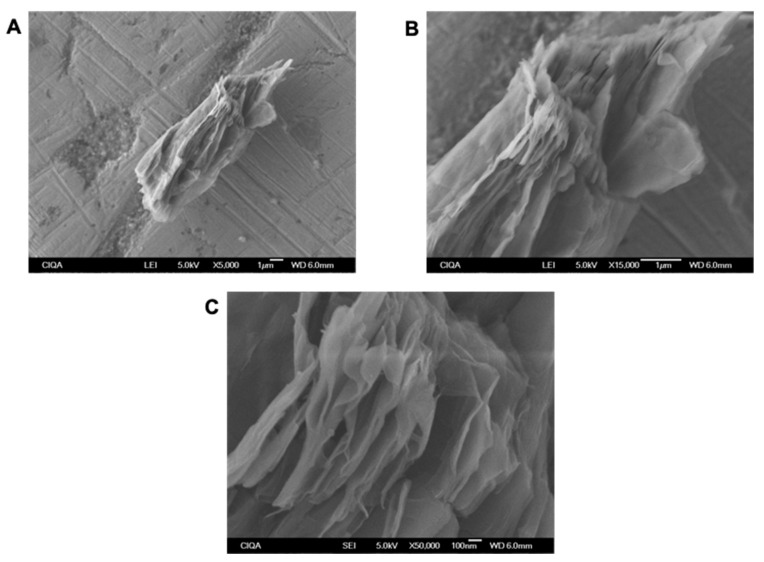
SEM micrographs of GrO at 5000× (**A**), 15,000× (**B**), and 50,000× (**C**).

SEM studies (Figure 6) of the nanohybrids indicate that, at 5000× (Figure 6A,D,G), a continuous, rugged surface made up of several layers is formed. However, no stacking of GO sheets is observed as in graphite where stacking is observed at lower magnifications (Figure 7). In rGO S, borders of the film created by the sheets (Figure 6B) are observed at 20,000 and 50,000× (Figure 6B,C), bonded by the covering starch (Figure 6C). The micrograph of rGO F at 20,000× (Figure 6E) shows a two-tone particle, representing GO (dark) and fructose (light). The grey tonalities are due to different electrical conductivities of the materials in rGO F. The material or area with the lowest electrical conductivity is charged with more electrons and, thus, its image is brighter. At 50,000× (Figure 6F), the nanohybrid surface shows borders representing GO sheets exposed on the surface. These observations were the result of low concentrations of grafted organic molecules onto rGO F (41.08% weight).

The rGO M micrograph at 20,000× (Figure 6H) shows no significant changes when compared against that at 5000× magnification (Figure 6G). Laminated surfaces are observed (Figure 6E) at 50,000×, but there are no differences in tonality indicating micro-cellulose and GO composition. SEM indicates that the number of organic molecules chemically grafted onto GO prevents them from auto assembling, as in graphite, since starch, fructose, and micro-cellulose hinder GO sheet stacking. Additionally, the volume of grafted molecules on these sheets (over 40% in weight) makes them bond to create nanometric and millimetric particles, observed as continuous and rugged surfaces in SEM.

The results demonstrate that sonochemistry promotes the chemical reaction between functional groups of graphite and starch as well as micro-cellulose and fructose molecules at reaction times of 30 min. However, the hybridization process is not the same for all organic molecules. As a background, it is known that ultrasound waves are applied in the chemical and thermal reduction of graphite. They have thermal and physical-mechanical effects, creating focal points with a high temperature (up to 5000 °K) and physical-mechanical changes, such as shear forces, micro-jets, and shock waves [68]. Therefore, ultrasound maximizes chemical reduction with chemical agents, such as hydrazine and graphite exfoliation, resulting in reduced and exfoliated graphene oxide. However, these thermal and physico-mechanical changes are affected by various parameters of the reaction medium, temperature, viscosity, and reagent concentration [69].

The TGA studies of nanohybrids rGO M and rGO F, (Figure 4B,C) show the characteristic thermal degradation behavior reported for micro-cellulose and fructose. However, the thermal degradation associated with the functional groups of graphite at 214 °C is not observed. Considering the above, nanohybrids do not show functional groups remaining from graphite. Similarly, the Raman spectroscopy studies of GO M and rGO F (Figure 3) show a ratio of I_D_/I_G_ intensities of 0.9736 and 1.0368 cm^−1^, respectively. The absolute difference with respect to graphite (0.9945 cm^−1^) is 0.0209 and 0.0423 cm^−1^, respectively. These values are not significant enough to suppose that fragmentation or regeneration of the hexagonal lattice of graphite carbon atoms occurs during the sonication process [70]. Therefore, these results indicate that the chemical reaction of the functional groups of graphite with micro-cellulose and fructose molecules prevails during the sonication process of rGO M and rGO F. In addition, no significant modification occurs in the molecular structure of the hexagonal lattice of carbon atoms of graphite.

In contrast, the TGA results of the starch nanohybrid (rGO S), (Figure 4A), indicate that some functional groups from graphite (residual groups) are still present after the sonication process, since it presents a signal thermal degradation at 218 °C. At the same time, it shows a characteristic thermal degradation signal of starch. These results indicate that not all the functional groups of graphite reacted with the molecules of the starch during the sonication process. Similarly, the Raman results of rGO S (Figure 3) show that its G band moves toward lower frequency values while the intensity of the D band decreases. Both behaviors occur when the functional groups of graphite are reduced, and the hexagonal network is re-established.

This behavior is due to the composition of the reaction mixture and the physicochemical characteristics of the starch. Since it is a bio-macromolecule, starch has a high molecular weight and presents polymorphism given that it has an amorphous and a crystalline fraction. During the synthesis process of the rGO S nanohybrid, the starch was initially solubilized in water and gelatinized before it was added to the graphite dispersion. In this gelatinization process, the starch granules absorb the water, swell, and lose their crystalline arrangement, increasing the viscosity of the medium unlike aqueous solutions of micro-cellulose and fructose. This increase in the viscosity generates two important effects in the sonication process: (I) The viscosity reduces the mobility of the particles during the sonication process [69], and (II) the disruption of the crystalline order of starch enhances the ability to absorb ultrasound waves and their energy. Due to the combination of both effects, not all the functional groups of graphite react with the starch, as shown in the TGA results (Figure 4A). They are not exposed efficiently, while the difficulty of the mobility of the reaction medium can cause some GO sheets to be directly exposed to the ultrasound waves and energy. This exposure removes some functional groups from the graphite, leaving the most stable part of the hexagonal network of carbon atoms. This partial reduction generates the displacement of the G band and the reduction in the D band.

## 4. Conclusions

The formation of chemically interlaced nanohybrids was proven through ultrasound energy between GO and starch, fructose, and micro-cellulose molecules at times under 30 min, unlike in literature reporting times of 9–72 h. This hybridization reaction does not significantly damage the GO structure since its I_D_/I_G_ ratio obtained by Raman spectroscopy does not show a high degree of chemical degradation.

The number of organic molecules grafted is over 40% the weight of the nanohybrids. This volume of grafting prevents the ordered stacking of GO layers in the nanohybrids, whose morphology was spherical, measuring from nanometers to millimeters. Furthermore, this degree of grafting is so significant that the organic molecules are crystallized on the GO sheets. This hybridization reaction is altered during the sonication of the reaction mixture since the viscosity of the reaction system promotes a partial reduction of GO, which occurred in rGO S. This effect was not observed in fructose nor micro-cellulose, since the GO functional groups fully react with these molecules. These results confirm that the nanohybrids rGO S, rGO F, and rGO M obtained show a high degree of exfoliation and hybridization, so they could be used in packaging films for food or medical implants. Future works will study the effect of the ultrasound energy applied to these reaction systems on the hybridization of the nanohybrids, changing the amplitude percentage and reaction time.

## Figures and Tables

**Table 1:** Functional groups and percentages obtained by X-ray photoelectron spectra (XPS) of graphite (GrO) and nanohybrids rGO S, rGO F, and rGO M.

Functional Group	Position (eV)/Concentration (%)							
	GrO		rGO S		rGO F		rGO M	
	eV	%	eV	%	eV	%	eV	%
**C–C and C=C**	284.7	46.8	284.5	14.3	284.8	16.0	284.7	4.5
**C–O**	286.3	10.2	-	-	-		-	
**C–O–C**	286.9	33.5	286.3	24.7	286.5	26.4	286.1	20.7
**C=O**	288.2	7.4	287.9	38.7	287.9	38.6	287.4	44.5
**O=C–OH**	289.4	2.1	288.8	16.9	289.3	10.4	288.6	22.4
**O=C–O**	-		290.1	5.3	290.7	8.5	289.8	7.8
**Relation % (C–C/C=O)**	6.3		0.4		0.4		0.1	

**Table 2:** Weight loss determined by TGA of rGO S, rGO F, and rGO M nanohybrids.

**Weight Loss**	**rGO S**			
	**Temperature (°C)**	**Assignation**	**Weight%**	**Maximum Peak (°C)**
1	40 a 150	Water	9.72	----
2	150–242	RGrO (OH, C–O–C)	9.23	218.1
3	242–450	SD	47.54	301.8
**Hybridization%**	47.54			
**% rGO**	33.51			
**Weight Loss**	**rGO F**			
	**Temperature (°C)**	**Assignation**	**Weight%**	**Maximum Peak (°C)**
1	40–145	Water	11.34	129.1
2	145–283	Fructose degradation	38.2	204.2
3	283–422		2.88	339.1
**Hybridization%**	41.08			
**% rGO**	47.58			
**Weight Loss**	**rGO M**			
	**Temperature (°C)**	**Assignation**	**Weight%**	**Maximum Peak (°C)**
1	120–267	TDH	13.36	230.1, 255.5
2	267–600	DC + DL	50.63	324.4, 446.8
**Hybridization%**	63.99			
**% rGO**	36.01			

DC: Decomposition Cellulose. DL: Degradation Lignin. RGrO: Residual Graphite. TDH: Thermal Depolymerization Hemicellulose. SD: Starch Degradation.

## Data Availability

The data presented in this study are available on request from the corresponding author.

## Supplementary Materials

The following are available online at https://www.mdpi.com/2073-4360/13/4/490/s1. Figure S1: Image of GO scattering before sonication (A) and nanohybrids after sonication (B). Figure S2: TGA thermograms of graphite (GrO) (A), starch (B), fructose (C), and microcellulose (D). Table S1: Weight loss assessed by TGA of graphite (GrO), starch, fructose, and micro-cellulose.

## References

[1] Castro Neto A.H., Guinea F., Peres N.M.R., Novoselov K.S., Geim A.K. (2009). The electronic properties of graphene. Rev. Mod. Phys..

[2] Huang X., Yin Z., Wu S., Qi X., He Q., Zhang Q., Yan Q., Boey F., Zhang H. (2011). Graphene-based materials: Synthesis, characterization, properties, and applications. Small.

[3] Sun X., Liu Z., Welsher K., Robinson J.T., Goodwin A., Zaric S., Dai H. (2008). Nano-graphene oxide for cellular imaging and drug delivery. Nano Res..

[4] Zhu Y., Murali S., Cai W., Li X., Suk J.W., Potts J.R., Ruoff R.S. (2010). Graphene and graphene oxide: Synthesis, properties, and applications. Adv. Mater..

[5] Wang Y., Polavarapu L., Liz-Marzán L.M. (2014). Reduced graphene oxide-supported gold nanostars for improved SERS sensing and drug delivery. ACS Appl. Mater. Interfaces.

[6] Valentini L., Cardinali M., Bittolo Bon S., Bagnis D., Verdejo R., Lopez-Manchado M.A., Kenny J.M. (2010). Use of butylamine modified graphene sheets in polymer solar cells. J. Mater. Chem..

[7] Ai W., Luo Z., Jiang J., Zhu J., Du Z., Fan Z., Xie L., Zhang H., Huang W., Yu T. (2014). Nitrogen and sulfur codoped graphene: Multifunctional electrode materials for high-performance LI-ion batteries and oxygen reduction reaction. Adv. Mater..

[8] Krishnan S.K., Singh E., Singh P., Meyyappan M., Nalwa H.S. (2019). A review on graphene-based nanocomposites for electrochemical and fluorescent biosensors. RSC Adv..

[9] Zhang Q., Xu J., Song Q., Li N., Zhang Z., Li K., Du Y., Wu L., Tang M., Liu L. (2014). Synthesis of amphiphilic reduced graphene oxide with an enhanced charge injection capacity for electrical stimulation of neural cells. J. Mater. Chem. B.

[10] Georgakilas V., Otyepka M., Bourlinos A.B., Chandra V., Kim N., Kemp K.C., Hobza P., Zboril R., Kim K.S. (2012). Functionalization of graphene: Covalent and non-covalent approach. Chem. Rev..

[11] Kuila T., Bose S., Mishra A.K., Khanra P., Kim N.H., Lee J.H. (2012). Chemical functionalization of graphene and its applications. Prog. Mater. Sci..

[12] Sedaghat E., Rostami A.A., Ghaemy M., Rostami A. (2019). Characterization, thermal degradation kinetics, and morphological properties of a graphene oxide/poly (vinyl alcohol)/starch nanocomposite. J. Therm. Anal. Calorim..

[13] Wu Z., Huang Y., Xiao L., Lin D., Yang Y., Wang H., Yang Y., Wu D., Chen H., Zhang Q. (2019). Physical properties and structural characterization of starch/polyvinyl alcohol/graphene oxide composite films. Int. J. Biol. Macromol..

[14] Golzari Aqda T., Behkami S., Raoofi M., Bagheri H. (2019). Graphene oxide-starch-based micro-solid phase extraction of antibiotic residues from milk samples. J. Chromatogr. A.

[15] Mohanty F., Swain S.K. (2019). Nano silver embedded starch hybrid graphene oxide sandwiched poly(ethylmethacrylate) for packaging application. Nano-Struct. Nano-Objects.

[16] Sharma C., Manepalli P.H., Thatte A., Thomas S., Kalarikkal N., Alavi S. (2017). Biodegradable starch/PVOH/laponite RD-based bionanocomposite films coated with graphene oxide: Preparation and performance characterization for food packaging applications. Colloid Polym. Sci..

[17] Amri A., Rahmana H., Utami S.P., Iriyanti R.S., Jiang Z.T., Rahman M.M. (2018). Conductive composites of tapioca based bioplastic and electrochemical-mechanical liquid exfoliation (emle) graphene. IOP Conf. Ser. Mater. Sci. Eng..

[18] Aqlil M., Moussemba Nzenguet A., Essamlali Y., Snik A., Larzek M., Zahouily M. (2017). Graphene Oxide Filled Lignin/Starch Polymer Bionanocomposite: Structural, Physical, and Mechanical Studies. J. Agric. Food Chem..

[19] Ferreira W.H., Dahmouche K., Andrade C.T. (2019). Tuning the mechanical and electrical conductivity properties of graphene-based thermoplastic starch/poly(lactic acid) hybrids. Polym. Compos..

[20] Li R., Liu C., Ma J. (2011). Studies on the properties of graphene oxide-reinforced starch biocomposites. Carbohydr. Polym..

[21] Luo D., Wang F., Vu B.V., Chen J., Bao J., Cai D., Willson R.C., Ren Z. (2018). Synthesis of graphene-based amphiphilic Janus nanosheets via manipulation of hydrogen bonding. Carbon.

[22] Usman A., Hussain Z., Riaz A., Khan A.N. (2016). Enhanced mechanical, thermal and antimicrobial properties of poly(vinyl alcohol)/graphene oxide/starch/silver nanocomposites films. Carbohydr. Polym..

[23] Chen Y., Dai G., Gao Q. (2019). Starch Nanoparticles-Graphene Aerogels with High Supercapacitor Performance and Efficient Adsorption. ACS Sustain. Chem. Eng..

[24] Htut Z., Kim M., Lee E., Lee G., Baeck S.H., Shim S.E. (2017). Biodegradable polymer-modified graphene/polyaniline electrodes for supercapacitors. Synth. Met..

[25] Wu W., Li Y., Yang L., Ma Y., Pan D., Li Y. (2014). A facile one-pot preparation of dialdehyde starch reduced graphene oxide/polyaniline composite for supercapacitors. Electrochim. Acta.

[26] Zhang X., Fan Q., Qu N., Yang H., Wang M., Liu A., Yang J. (2019). Ultrathin 2D nitrogen-doped carbon nanosheets for high performance supercapacitors: Insight into the effects of graphene oxides. Nanoscale.

[27] Franco A., Cano M., Giner-Casares J.J., Rodríguez-Castellón E., Luque R., Puente-Santiago A.R. (2019). Boosting the electrochemical oxygen reduction activity of hemoglobin on fructose@graphene-oxide nanoplatforms. Chem. Commun..

[28] Bhattacharyya A., Banerjee B., Ghorai S., Rana D., Roy I., Sarkar G., Saha N.R., De S., Ghosh T.K., Sadhukhan S. (2018). Development of an auto-phase separable and reusable graphene oxide-potato starch based cross-linked bio-composite adsorbent for removal of methylene blue dye. Int. J. Biol. Macromol..

[29] Hosseinzadeh H., Ramin S. (2016). Fast and enhanced removal of mercury from aqueous solutions by magnetic starch-g-poly(acryl amide)/graphene oxide nanocomposite superabsorbents. Polym. Sci. Ser. B.

[30] Lou Z., Cao Z., Xu J., Zhou X., Zhu J., Liu X., Ali Baig S., Zhou J., Xu X. (2017). Enhanced removal of As(III)/(V) from water by simultaneously supported and stabilized Fe-Mn binary oxide nanohybrids. Chem. Eng. J..

[31] Jodar L.V., Santos F.A., Zucolotto V., Janegitz B.C. (2018). Electrochemical sensor for estriol hormone detection in biological and environmental samples. J. Solid State Electrochem..

[32] Li D., Duan H., Ma Y., Deng W. (2018). Headspace-Sampling Paper-Based Analytical Device for Colorimetric/Surface-Enhanced Raman Scattering Dual Sensing of Sulfur Dioxide in Wine. Anal. Chem..

[33] Wang Y., Huang F., Chen X., Wang X.W., Zhang W.B., Peng J., Li J., Zhai M. (2018). Stretchable, Conductive, and Self-Healing Hydrogel with Super Metal Adhesion. Chem. Mater..

[34] Wu D., Samanta A., Srivastava R.K., Hakkarainen M. (2017). Starch-Derived Nanographene Oxide Paves the Way for Electrospinnable and Bioactive Starch Scaffolds for Bone Tissue Engineering. Biomacromolecules.

[35] Liu K., Wang Y., Li H., Duan Y. (2015). A facile one-pot synthesis of starch functionalized graphene as nano-carrier for pH sensitive and starch-mediated drug delivery. Colloids Surf. B Biointerfaces.

[36] Mohan V.B., Jayaraman K., Bhattacharyya D. (2018). Fabrication of highly conductive graphene particle-coated fiber yarns using polymeric binders through efficient coating techniques. Adv. Polym. Technol..

[37] Yarahmadi E., Didehban K., Shabanian M., Saeb M.R. (2018). High-performance starch-modified graphene oxide/epoxy nanocomposite coatings: A glimpse at cure kinetics and fracture behavior. Prog. Color Color. Coat..

[38] Zheng P., Ma T., Ma X. (2013). Fabrication and properties of starch-grafted graphene nanosheet/plasticized-starch composites. Ind. Eng. Chem. Res..

[39] Sandhya P.K., Sreekala M.S., Padmanabhan M., Jesitha K., Thomas S. (2019). Effect of starch reduced graphene oxide on thermal and mechanical properties of phenol formaldehyde resin nanocomposites. Compos. Part B Eng..

[40] Soleimani K., Dadkhah Tehrani A., Adeli M., Sattari S. (2018). Convenient method for preparation of a new absorbent based on biofunctionalized graphene oxide hydrogels using nitrene chemistry and click reaction. Iran. Polym. J..

[41] Srivastava J., Kushwaha A., Srivastava M., Srivastava A., Singh M. (2019). Glycoprotein imprinted RGO-starch nanocomposite modified EQCM sensor for sensitive and specific detection of transferrin. J. Electroanal. Chem..

[42] Srivastava J., Kushwaha A., Singh M. (2018). Imprinted Graphene-Starch Nanocomposite Matrix-Anchored EQCM Platform for Highly Selective Sensing of Epinephrine. Nano.

[43] Teng J., Zeng X., Xu X., Yu J.G. (2018). Assembly of a novel porous 3D graphene oxide-starch architecture by a facile hydrothermal method and its adsorption properties toward metal ions. Mater. Lett..

[44] Wang Z., Zhang X., Wu X., Yu J.G., Jiang X.Y., Wu Z.L., Hao X. (2017). Soluble starch functionalized graphene oxide as an efficient adsorbent for aqueous removal of Cd(II): The adsorption thermodynamic, kinetics and isotherms. J. Sol. Gel. Sci. Technol..

[45] Wu D., Bäckström E., Hakkarainen M. (2017). Starch Derived Nanosized Graphene Oxide Functionalized Bioactive Porous Starch Scaffolds. Macromol. Biosci..

[46] Yarahmadi E., Didehban K., Sari M.G., Saeb M.R., Shabanian M., Aryanasab F., Zarrintaj P., Paran S.M.R., Mozafari M., Rallini M. (2018). Development and curing potential of epoxy/starch-functionalized graphene oxide nanocomposite coatings. Prog. Org. Coat..

[47] Hernández-Hernández E., Hernández-Belmares P.J., Ceniceros-Reyes M.A., Rodríguez-Fernández O.S., González-Morones P. (2016). Graphite Oxide: A Simple and Reproducible Synthesis Route. Graphene Production and Application.

[48] Krishnamoorthy K., Veerapandian M., Yun K., Kim S.J. (2013). The chemical and structural analysis of graphene oxide with different degrees of oxidation. Carbon.

[49] Ma T., Chang P.R., Zheng P., Ma X. (2013). The composites based on plasticized starch and graphene oxide/reduced graphene oxide. Carbohydr. Polym..

[50] Wu L.M., Tong D.S., Zhao L.Z., Yu W.H., Zhou C.H., Wang H. (2014). Fourier transform infrared spectroscopy analysis for hydrothermal transformation of microcrystalline cellulose on montmorillonite. Appl. Clay Sci..

[51] Ibrahim M., Alaam M., El-Haes H., Jalbout A.F., De Leon A. (2006). Analysis of the structure and vibrational spectra of glucose and fructose. Eclet. Quim..

[52] Kizil R., Irudayaraj J., Seetharaman K. (2002). Characterization of irradiated starches by using FT-Raman and FTIR spectroscopy. J. Agric. Food Chem..

[53] Brenner T., Kiessler B., Radosta S., Arndt T. (2016). Processing surface sizing starch using oxidation, enzymatic hydrolysis and ultrasonic treatment methods—Preparation and application. Carbohydr. Polym..

[54] Wei B., Qi H., Wang Z., Bi Y., Zou J., Xu B., Ren X., Ma H. (2020). The ex-situ and in-situ ultrasonic assisted oxidation of corn starch: A comparative study. Ultrason. Sonochem..

[55] Ambre J.P., Dhopte K.B., Nemade P.R., Dalvi V.H. (2019). High flux hyperbranched starch-graphene oxide piperazinamide composite nanofiltration membrane. J. Environ. Chem. Eng..

[56] Acik M., Lee G., Mattevi C., Pirkle A., Wallace R.M., Chhowalla M., Cho K., Chabal Y. (2011). The role of oxygen during thermal reduction of graphene oxide studied by infrared absorption spectroscopy. J. Phys. Chem. C.

[57] O’Connell C. (1999). The effects of methylparaben on the gelatinization and thermal decomposition of corn starch. Thermochim. Acta.

[58] Abdolmaleki A., Mallakpour S., Rostami M. (2015). Performance evaluation of fructose-functionalized multiwalled carbon nanotubes/biodegradable poly(amide-imide) based on N, N′ -(pyromellitoyl)-bis- S -valine bionanocomposite. High Perform. Polym..

[59] Mallakpour S., Abdolmaleki A., Rostami M. (2014). Morphology and thermal properties of environmental friendly nanocomposites using biodegradable poly(amide–imide) based on N-trimellitylimido-S-valine matrix reinforced by fructose-functionalized multi-walled carbon nanotubes. Colloid Polym. Sci..

[60] Mallakpour S., Behranvand V. (2015). Sonochemical production and characterization of d-fructose functionalized MWCNTs/alanine-based poly(amide-imide) nanocomposites. Colloid Polym. Sci..

[61] Mallakpour S., Madani M. (2016). Synthesis, structural characterization, and tensile properties of fructose functionalized multi-walled carbon nanotubes/chitosan nanocomposite films. J. Plast. Film Sheeting.

[62] Mallakpour S., Behranvand V. (2016). Improved solubilization of multiwalled carbon nanotubes (MWCNTs) in water by surface functionalization with d -glucose and d fructose: Properties comparison of functionalized MWCNTs/alanine-based poly(amide-imide) nanocomposites. High Perform. Polym..

[63] Khenblouche A., Bechki D., Gouamid M., Charradi K., Segni L., Hadjadj M., Boughali S. (2019). Extraction and characterization of cellulose microfibers from Retama raetam stems. Polimeros.

[64] Guinesi L.S., da Róz A.L., Corradini E., Mattoso L.H.C., de Teixeira E.M., Curvelo A.A.D.S. (2006). Kinetics of thermal degradation applied to starches from different botanical origins by non-isothermal procedures. Thermochim. Acta.

[65] Manek R.V., Builders P.F., Kolling W.M., Emeje M., Kunle O.O. (2012). Physicochemical and binder properties of starch obtained from Cyperus esculentus. AAPS PharmSciTech.

[66] Rotaru P., Scorei R., Hrbor A., Dumitru M.D. (2010). Thermal analysis of a calcium fructoborate sample. Thermochim. Acta.

[67] Gong J., Li J., Xu J., Xiang Z., Mo L. (2017). Research on cellulose nanocrystals produced from cellulose sources with various polymorphs. RSC Adv..

[68] Abulizi A., Okitsu K., Zhu J.J. (2014). Ultrasound assisted reduction of graphene oxide to graphene in l-ascorbic acid aqueous solutions: Kinetics and effects of various factors on the rate of graphene formation. Ultrason. Sonochem..

[69] Jain A., Kaur P., Juglan K.C. (2020). Synthesis and Ultrasonic Investigation of Reduced Graphene Oxide Nanosuspension with Water. J. Phys. Conf. Ser..

[70] Narayanan K.B., Park G.T., Han S.S. (2020). Antibacterial properties of starch-reduced graphene oxide–polyiodide nanocomposite. Food Chem..

