# Supplementary material for: Covalent Functionalization of Graphene Oxide with Fructose, Starch, and Micro-Cellulose by Sonochemistry
Source: Polymers (Basel). 2021 Feb 4;13(4):490. doi: 10.3390/polym13040490 (PMC7915305; doi:10.3390/polym13040490)
Supplement: Supplementary file 1 [file polymers-13-00490-s001.pdf]

Supplementary information:

Covalent functionalization of graphene oxide with fructose, starch, and microcellulose by sonochemistry.

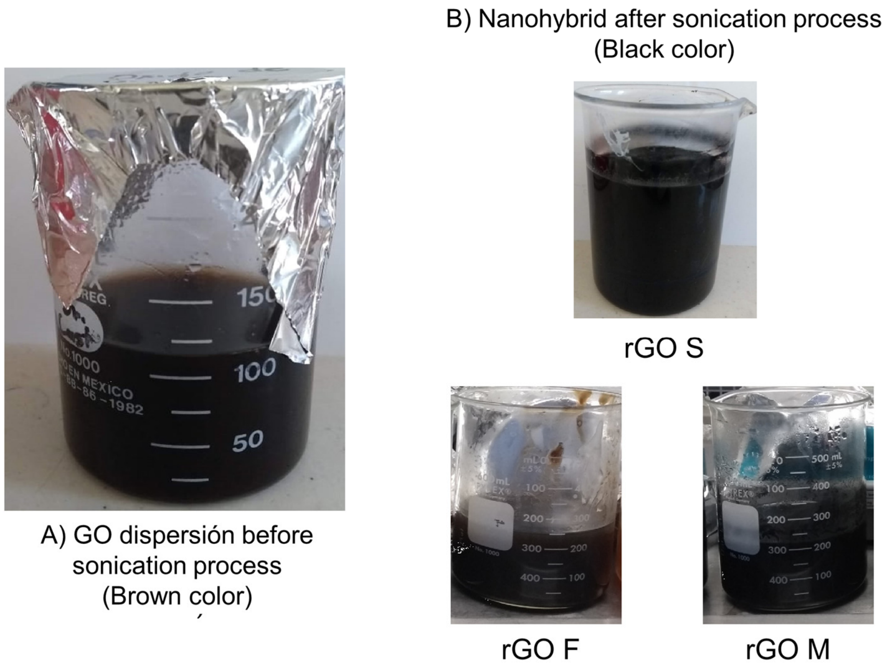

Figure S1. Image of GO scattering before sonication (A) and nanohybrids after sonication (B).

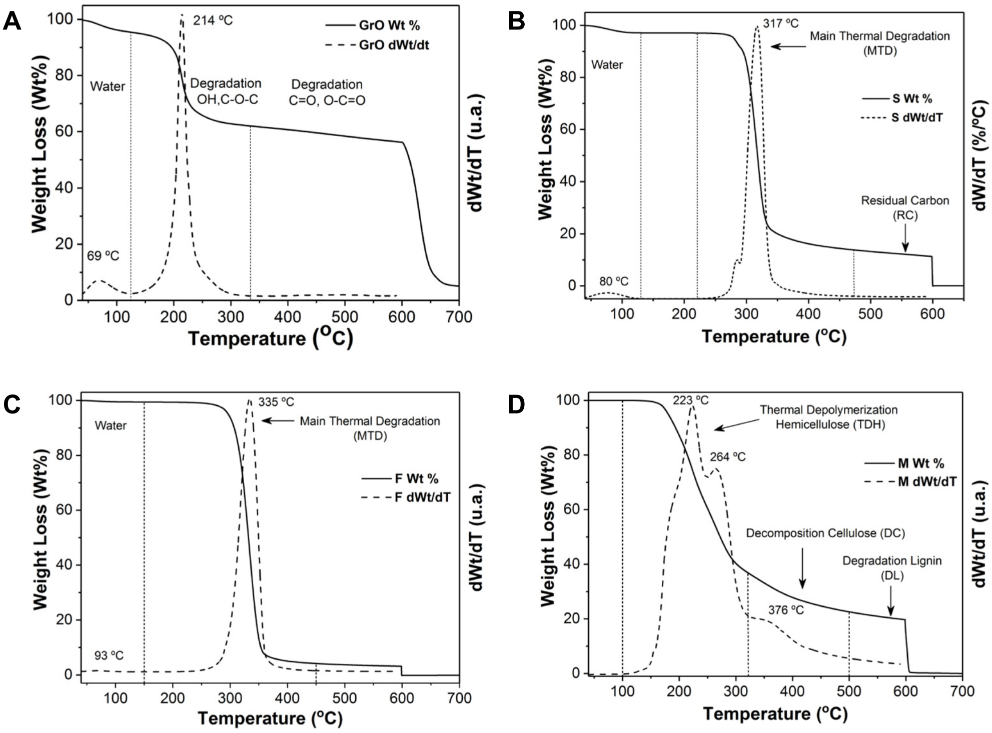

Figure S2. TGA thermograms of graphite (GrO) (A), starch (B), fructose (C), and microcellulose (D).

Table S1. Weight loss assessed by TGA of graphite (GrO), starch, fructose, and microcellulose.

Weight loss	**GrO** [1]			
	**Temperature (°C)**	**Assignment**	**Weight %**	**Maximum peak (°C)**
1	40 a 120	Water	4.58	68.4
2	120–335	OH, C–O–C	33.48	214.7
3	335–600	C=O, O–C=O	5.65	----
**Weight loss**	**Starch** [2]			
	**Temperature (°C)**	**Assignment**	**Weight %**	**Maximum peak (°C)**
1	40–130	Water	3.08	80
2	220–472	MTD	83.1	317.4
3	472–600	RC	2.88	----
**Weight loss**	**Fructose**			
	**Temperature (°C)**	**Assignment**	**Weight %**	**Maximum peak (°C)**
1	40–150	Water	0.66	----
2	150–450	MTD	95.09	335.1
**Weight loss**	**Microcellulose** [3]			
	**Temperature (°C)**	**Assignment**	**Weight %**	**Maximum peak (°C)**
1	100–321	TDH	63.81	223.5, 264.5
2	321–500	DC	14.3	376.6
3	500–600	DL	2.98	----

MTD: Main Thermal Degradation. RC: Residual Carbon. DL: Degradation of Lignin. TDH: Thermal Depolymerization Hemicellulose. DC: Decomposition Cellulose.

References

[1] M. Acik, G. Lee, C. Mattevi, A. Pirkle, R.M. Wallace, M. Chhowalla, K. Cho, Y. Chabal, The role of oxygen during thermal reduction of graphene oxide studied by infrared absorption spectroscopy, J. Phys. Chem. C. 115 (2011) 19761–19781. https://doi.org/10.1021/jp2052618.

[2] C. O'Connell, The effects of methylparaben on the gelatinization and thermal decomposition of corn starch, Thermochim. Acta. 340–341 (1999) 183–194. https://doi.org/10.1016/S0040-6031(99)00263-4.

[3] A. Khenblouche, D. Bechki, M. Gouamid, K. Charradi, L. Segni, M. Hadjadj, S. Boughali, Extraction and characterization of cellulose microfibers from Retama raetam stems, Polimeros. 29 (2019) 1–8. https://doi.org/10.1590/0104-1428.05218.
